# Association of Postsurgical Opioid Refills for Patients With Risk of Opioid Misuse and Chronic Opioid Use Among Family Members

**DOI:** 10.1001/jamanetworkopen.2022.21316

**Published:** 2022-07-15

**Authors:** Denis Agniel, Gabriel A. Brat, Jayson S. Marwaha, Kathe Fox, Daniel Knecht, Harold L. Paz, Mark C. Bicket, Brian Yorkgitis, Nathan Palmer, Isaac Kohane

**Affiliations:** 1 RAND Corporation, Santa Monica, California; 2 Department of Biomedical Informatics, Harvard Medical School, Boston, Massachusetts; 3 Department of Surgery, Beth Israel Deaconess Medical Center, Boston, Massachusetts; 4 Aetna Inc, Hartford, Connecticut; 5 Johns Hopkins University School of Medicine, Baltimore, Maryland; 6 Johns Hopkins Bloomberg School of Public Health, Baltimore, Maryland; 7 Department of Surgery, University of Florida, Jacksonville

## Abstract

**Importance:**

The US health care system is experiencing a sharp increase in opioid-related adverse events and spending, and opioid overprescription may be a key factor in this crisis. Ambient opioid exposure within households is one of the known major dangers of overprescription.

**Objective:**

To quantify the association between the postsurgical initiation of prescription opioid use in opioid-naive patients and the subsequent prescription opioid misuse and chronic opioid use among opioid-naive family members.

**Design, Setting, and Participants:**

This cohort study was conducted using administrative data from the database of a US commercial insurance provider with more than 35 million covered individuals. Participants included pairs of patients who underwent surgery from January 1, 2008, to December 31, 2016, and their family members within the same household. Data were analyzed from January 1 to November 30, 2018.

**Exposures:**

Duration of opioid exposure and refills of opioid prescriptions received by patients after surgery.

**Main Outcomes and Measures:**

Risk of opioid misuse and chronic opioid use in family members were calculated using inverse probability weighted Cox proportional hazards regression models.

**Results:**

The final cohort included 843 531 pairs of patients and family members. Most pairs included female patients (445 456 [52.8%]) and male family members (442 992 [52.5%]), and a plurality of pairs included patients in the 45 to 54 years age group (249 369 [29.6%]) and family members in the 15 to 24 years age group (313 707 [37.2%]). A total of 3894 opioid misuse events (0.5%) and 7485 chronic opioid use events (0.9%) occurred in family members. In adjusted models, each additional opioid prescription refill for the patient was associated with a 19.2% (95% CI, 14.5%-24.0%) increase in hazard of opioid misuse in family members. The risk of opioid misuse appeared to increase only in households in which the patient obtained refills. Family members in households with any refill had a 32.9% (95% CI, 22.7%-43.8%) increased adjusted hazard of opioid misuse. When patients became chronic opioid users, the hazard ratio for opioid misuse among family members was 2.52 (95% CI, 1.68-3.80), and similar patterns were found for chronic opioid use.

**Conclusions and Relevance:**

This cohort study found that opioid exposure was a household risk. Family members of a patient who received opioid prescription refills after surgery had an increased risk of opioid misuse and chronic opioid use.

## Key Points

**Question:**

What is the association between a patient’s new opioid exposure after hospital discharge and subsequent opioid misuse and chronic opioid use in family members in the same household?

**Findings:**

In this cohort study of 843 531 patient and family member pairs, each additional opioid prescription refill for the patients was associated with increased hazard of opioid misuse among family members in adjusted models. The risk of opioid misuse and chronic opioid use increased in households in which the patient obtained refills.

**Meaning:**

Findings of this study suggest that family members in a household with a patient who received opioid prescription refills may have an increased risk of opioid misuse and chronic opioid use.

## Teaser

This cohort study examines the association between the receipt of opioid prescriptions after surgery and the risk of opioid misuse and chronic opioid use among family members of the patient.

## Introduction

For the past several years, the US health care system has experienced a sharp increase in opioid overdose deaths,^1^ nonfatal overdoses,^2^ opioid-related emergency department visits,^3^ and spending on opioid treatment.^4^ This pattern has been further exacerbated by the COVID-19 pandemic. By many metrics, such as opioid overdose deaths and opioid misuse events, the opioid crisis has reached historic levels in the US,^5^ making it increasingly important to understand the risk factors for opioid-related adverse events.

Opioid overprescription after surgery may be a key factor in the opioid crisis given that patients who undergo surgery are 3 times more likely to get an opioid prescription than those who do not, whereas postoperative discharge prescriptions across all surgical specialties far exceed the actual requirement for appropriate pain control.^6,7,8,9,10,11^ Between 67% and 92%^6,7,8,9,10^ of patients who have surgery have leftover opioids, and 42% to 71% of pills are left unused after surgery.^11^ Longer durations and higher total doses of these postsurgical prescriptions are associated with the risk of subsequent opioid misuse.^12^

Evidence suggests that ambient exposure to opioids within households confers risks as well, even on individuals who were not directly prescribed the opioids. One of many purported mechanisms underlying these risks is the diversion of prescribed pills: an estimated 65% to 80% of those with prescription opioid misuse report a source that is not a prescription in their name.^13,14^ Although such diversion occurs through many channels, the most common sources are family members and friends.^13,14^ An estimated 55% of all individuals who misuse opioids report obtaining prescription opioids from family and friends,^13,15^ whereas 22% describe taking opioids from friends or relatives without asking.^16^ Indeed, ease of access within the household enables diversion: 73% to 77% of patients leave their unused opioids unlocked and accessible.^11^ Furthermore, 82% of these unused opioids are prescribed by a single physician.^13^

In addition to being harmful on its own, misuse of prescription opioids appears to be a gateway to use of illicit opioids.^17^ An estimated 4% to 6% of those who misuse prescription opioids transition to heroin use.^18,19,20^ Those with opioid use disorder are 40 times more likely to also use heroin.^21^ Among new users of heroin, 66% report misusing prescription opioids before starting heroin.^22^

In recent years, there have been attempts to measure the harmful downstream consequences of household opioid exposure.^23,24,25,26,27,28^ One large retrospective study of claims data found that, among those with opioid use disorder without their own prescription, 51% had access to opioids through a household member’s prescription.^29^ Another study found that prescription opioid use by a patient in the household increased another member’s absolute risk of starting prescription opioids by 0.7%.^30^ Furthermore, other studies found that family prescription opioid use is associated with increased odds of opioid misuse^23^ as well as overdose^25^ among adolescents. However, how households of patients fare given their heightened exposure to postsurgical prescription opioids remains unknown.

In this study, we sought to quantify the association between the postsurgical initiation of prescription opioid use in opioid-naive patients and the subsequent prescription opioid misuse and chronic opioid use among opioid-naive family members. We specifically evaluated 2 outcome measures in family members: (1) chronic opioid use as measured by filled opioid prescriptions and (2) opioid misuse events as measured by downstream diagnosis codes. These outcomes captured complementary, common measures of opioid misuse from different data sources.

## Methods

This cohort study was approved by the institutional review board at Harvard Medical School, which waived the requirement for informed consent because the data were deidentified and only aggregated results were reported. We followed the Strengthening the Reporting of Observational Studies in Epidemiology (STROBE) reporting guideline.

### Data Source and Cohort Selection

Patients and their families were selected from a deidentified administrative database of Aetna Inc, a commercial managed health care company. This database stores data for more than 35 million individuals with Aetna health and pharmacy insurance coverage from January 1, 2008, to December 31, 2016. Employee subscribers and their dependents were defined by a unique subscriber identifier. Data included all medical and pharmacy claims during the study period. Race and ethnicity data were not collected in this study because our goal was to identify household risks of opioids at the population level. In addition, race and ethnicity data were not available in the deidentified database.

Patients were identified as those who filed claims for inpatient and outpatient surgical procedures with *Current Procedural Terminology, Fourth Edition*, codes that are included in a comprehensive list released by the National Surgical Quality Improvement Program of the American College of Surgeons in 2015.^21^ The network of each patient consisted of those who shared the same subscriber identifier as the patient. This network may include spouses, children, and parents as well as (rarely) those with other relationships with the patient (Table). For this reason, in this study, we referred to individuals in a patient’s network as family members and to the network itself as a household, although this was a misnomer in some cases. Either the patient or a family member (or neither) may be the employee subscriber. For analysis, each family member was categorized into employee, spouse (of employee), child (of employee), or other relationship.

**Table.** Basic Characteristics of the Patient and Family Member Pairs^a^

Characteristic	No. (%)			
	Total	Household with 0 opioid prescriptions	Household with 1 opioid prescription	Household with any opioid prescription refill
All pairs	843 531 (100)	303 787 (36.0)	397 418 (47.1)	142 326 (16.9)
Family member sex				
Female	400 539 (47.5)	140 890 (46.4)	190 874 (48.0)	68 775 (48.3)
Male	442 992 (52.5)	162 897 (53.6)	206 544 (52.0)	73 551 (51.7)
Patient sex				
Female	445 456 (52.8)	166 194 (54.7)	204 347 (51.4)	74 915 (52.6)
Male	398 075 (47.2)	137 593 (45.3)	193 071 (48.6)	67 411 (47.4)
Family member age, y				
15-24	313 707 (37.2)	108 603 (35.7)	149 345 (37.6)	55 759 (39.2)
25-34	61 942 (7.3)	23 861 (7.9)	29 021 (7.3)	9060 (6.4)
35-44	151 847 (18.0)	58 469 (19.2)	72 016 (18.1)	21 362 (15.0)
45-54	199 449 (23.6)	70 248 (23.1)	95 379 (24.0)	33 822 (23.8)
55-64	116 586 (13.8)	42 606 (14.0)	51 657 (13.0)	22 323 (15.7)
Patient age, y				
0-14	94 118 (11.2)	46 503 (15.3)	42 959 (10.8)	4656 (3.3)
15-24	168 544 (20.0)	52 842 (17.4)	84 751 (21.3)	30 951 (21.7)
25-34	41 793 (5.0)	13 683 (4.5)	20 235 (5.1)	7875 (5.5)
35-44	141 735 (16.8)	47 817 (15.7)	67 656 (17.0)	26 262 (18.5)
45-54	249 369 (29.6)	88 289 (29.1)	116 847 (29.4)	44 233 (31.1)
55-64	132 272 (15.7)	48 464 (16.0)	58 365 (14.7)	25 443 (17.9)
65-94	15 700 (1.9)	6189 (2.0)	6605 (1.7)	2906 (2.0)
Family member relationship to insurance subscriber				
Child	320 387 (38.0)	110 740 (36.5)	152 497 (38.4)	57 150 (40.2)
Employee	285 422 (33.8)	107 495 (35.4)	131 145 (33.0)	46 782 (32.9)
Spouse	234 470 (27.7)	84 414 (27.8)	112 326 (28.3)	37 730 (26.5)
Other^b^	3252 (0.4)	1138 (0.4)	1450 (0.4)	664 (0.5)
Patient relationship to insurance subscriber				
Child	264 296 (31.3)	99 883 (32.9)	128 378 (32.3)	36 035 (25.3)
Employee	293 192 (34.8)	99 102 (32.6)	140 559 (35.4)	53 531 (37.6)
Spouse	282 693 (33.5)	103 614 (34.1)	127 043 (32.0)	52 036 (36.6)
Other^b^	3350 (0.4)	1188 (0.4)	1438 (0.4)	724 (0.5)

^a^ Overall, 525 524 patients and 772 611 family members were included, resulting in 843 531 unique pairs of patients and family members. Some patients had multiple family members, and some family members were associated with multiple patients.

^b^ Other relationship included dependent, adult relative, or parent.

To be included in the study, family members were required to be between ages 15 and 64 years and to satisfy the same inclusion criteria as the patients: 6 months of medical and 3 months of pharmacy coverage before the surgical date as well as 12 months of medical and 3 months of pharmacy coverage after the surgical date. We required both the family members and the patients to exhibit no evidence of opioid or related misuse in the 6 months before surgery. We defined presurgical opioid misuse as the presence of a claim with an *International Statistical Classification of Diseases, Tenth Revision, Clinical Modification* (*ICD-10-CM*) diagnosis code for opioid dependence, abuse, or overdose (eTable 2 in the Supplement) within 6 months before surgery. We required both the patients and family members to be opioid naive, with a prescription fill of 7 or fewer days’ supply of opioids within 60 days before surgery for the patients and 0 days’ supply for the family members.^12,31^

### Opioid Exposure

Postsurgical opioid exposure was measured as all opioid prescriptions filled by the patient in the 90 days after surgery. Opioids were identified by therapeutic category using the Multum Lexicon Drug Database (Cerner Corporation).^32^ We considered the following 5 measures of opioid exposure for the patient: indication of any opioid prescription (any opioid), total weeks’ supply of postsurgical opioids (duration), number of prescriptions after the initial postdischarge prescription (number of refills), more than 1 additional opioid prescription (any refill), and longer than 90 days’ supply (long-term exposure). For the purposes of computationally efficient analysis, duration of exposure was truncated at 6 weeks and number of refills was truncated at 5.

### Outcome Measures

We measured 2 outcomes in family members: opioid misuse and chronic opioid use. Opioid misuse and chronic opioid use were measured in the subsequent 12 months after the date of surgery in the patient.

We defined opioid misuse as the presence of a claim with an *ICD-10-CM* diagnosis code for opioid dependence, abuse, or overdose (eTable 1 in the Supplement) in the 12-month follow-up period after patient surgery. This composite end point, established a priori according to previous and related work,^12,33^ encompassed a select group of forms of misuse related specifically to opioid misuse and poisoning. Only *ICD-10-CM* diagnosis codes related specifically to prescription opioids were included.

We defined chronic opioid use as an opioid use episode longer than 90 days, as measured by filled opioid prescriptions with no more than 30 days between successive refills. This outcome and threshold are commonly used in the literature to identify chronic opioid use.^31,34,35^

### Statistical Analysis

The 95% CIs for raw rates of opioid misuse and chronic opioid use were computed using the exponential survival model. To calculate adjusted estimates of rates of opioid misuse and chronic opioid use, we used inverse probability of treatment weighted Cox proportional hazards regression models to assess the implication of all opioid exposures.

All prescription drugs and diagnoses that occurred in at least 0.05% of patients and family members in the presurgical period were considered as covariates. We then used penalized logistic regression (Lasso)^36^ to screen the presurgical diagnoses and drugs of interest for covariate adjustment, which included sex of patients and family members, year of surgery, presurgical diagnoses for patients and family members, and quantity and duration of classes of prescription drugs. Lasso propensity score models were estimated using all covariates from the initial screening models. Propensity scores were used to calculate the inverse probability of treatment weights that were incorporated into the Cox proportional hazards regression models, from which we estimated hazard ratios (HRs). Models included the exposure of interest and any covariates from the propensity score model that were found to be imbalanced after weighting (eTables 17 and 18 in the Supplement). No interactions were considered. Censoring occurred in these models because of loss or change of coverage under the same insurer, which may occur owing to death, health plan disenrollment, or changes in insurance coverage.

Age-specific associations with opioid misuse and chronic opioid use were estimated using weighted generalized additive models. Generalized additive models are a type of generalized linear model that can be fit to complex nonlinear relationships while retaining sufficient interpretability to estimate the associations between variables.^37^ Generalized additive models were weighted in a similar fashion as the Cox proportional hazards regression models to perform covariate adjustment. The eMethods in the Supplement provides power calculations and more statistical details.

An α = .05 was considered statistically significant. All analyses were 2-tailed and performed in R, version 3.3.1 (R Foundation for Statistical Computing), and were conducted from January 1 to November 30, 2018.

## Results

A total of 525 524 patients and 772 611 family members were included in this study, resulting in 843 531 unique pairs of patient and family member. Most pairs included female patients (445 456 [52.8%]) and male family members (442 992 [52.5%]), and a plurality of pairs included patients in the 45 to 54 years age group (249 369 [29.6%]) and family members in the 15 to 24 years age group (313 707 [37.2%]) (Table). This cohort was derived from a total of 772 676 patients with other covered family members in their household who satisfied the requirements for insurance coverage. Patients (identified as those who underwent surgery) were associated with a total of 1 308 312 family members, for a total of 1 801 164 patient and family member pairs; some patients had multiple family members, and some family members were associated with multiple patients. Exclusions were made for family member enrollment, age, opioid misuse diagnosis, and presurgical opioid use; eFigure in the Supplement provides a full breakdown of exclusions.

In 47.1% of pairs (n = 397 418), the patients received only 1 initial opioid prescription, whereas 36.0% of pairs (n = 303 787) were in households with 0 opioid prescriptions. In 16.9% of pairs (n = 142 326), the patients obtained at least 1 refill. Family member characteristics were not generally associated with receipt of opioids by the patient.

A total of 3894 opioid misuse (0.5%) and 7485 chronic opioid use events (0.9%) were identified in family members (rates of opioid misuse in the index patient can be found in a previous work^29^). Among the 303 787 pairs in which the patients received 0 opioid prescriptions, there were 1210 opioid misuse events (0.4%; 131 per 100 000 person-years) and 2253 chronic opioid use events (0.7%; 245 per 100 000 person-years). In 539 744 pairs within opioid-exposed households, there were 2684 opioid misuse events (0.5%; 163 per 100 000 person-years) and 5232 chronic opioid use events (1.0%; 318 per 100 000 person-years) (eTables 3 and 4 in the Supplement). When the patient was exposed to at least 5 opioid prescriptions (n = 7942), the incidence of opioid misuse in the household increased to 1.3% (95% CI, 1.0%-1.5%), with a rate of 428 per 100 000 person-years (95% CI, 345 to 511 per 100 000 person-years), and the incidence of chronic opioid use increased to 2.4% (95% CI, 2.1%-2.8%), with a rate of 817 per 100 000 person-years (95% CI, 702 to 932 per 100 000 person-years) (eTables 7 and 8 in the Supplement). The eTables 5 to 16 in the Supplement provide details of the incidence of opioid misuse and chronic opioid use in family members by the patient’s duration of opioid exposure, number of refills, and long-term exposure.

**Figure 1.**
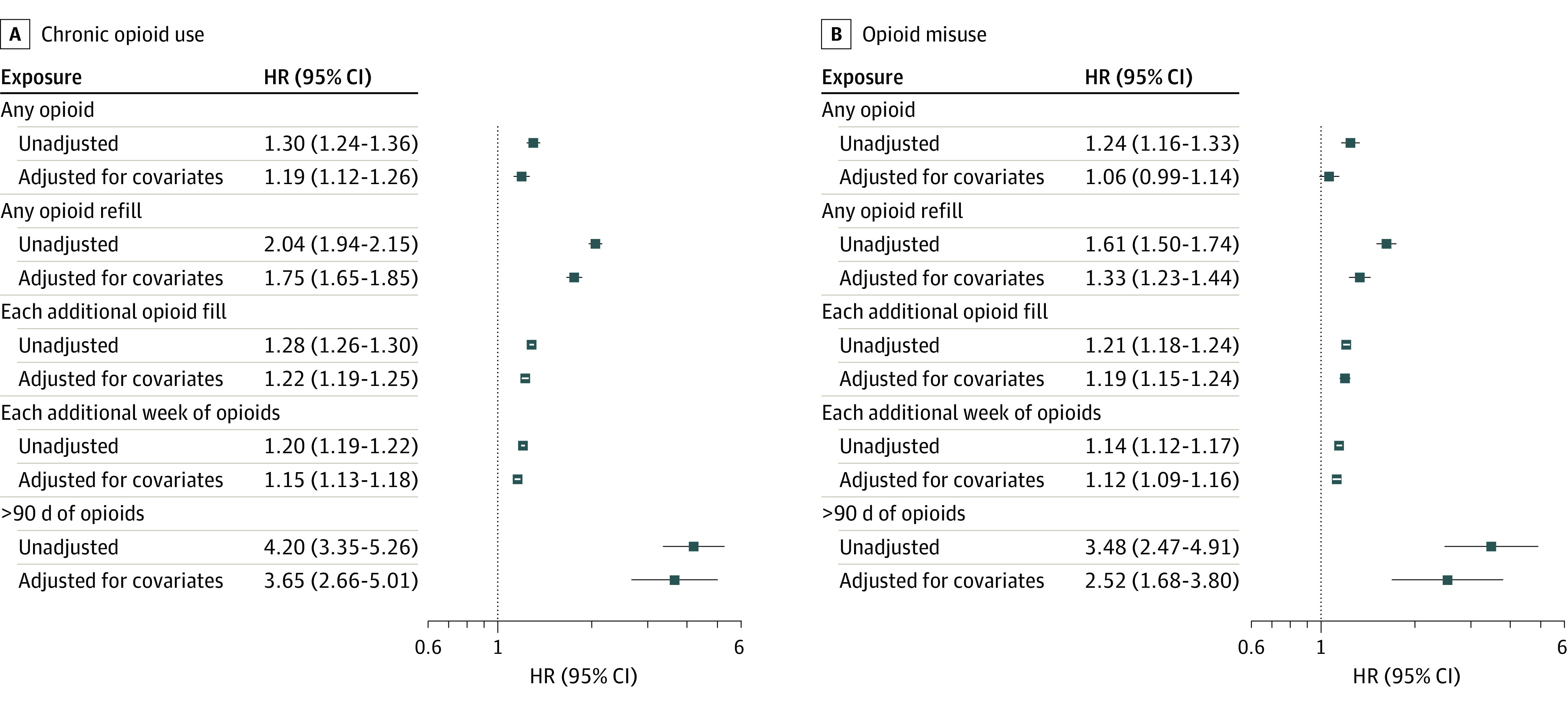
Hazard Ratios for Associations Between All Opioid Exposures in Patients and Opioid Misuse and Chronic Opioid Use in Family Members HR indicates hazard ratio.

Family members in the household of a patient who received any opioid prescription had an adjusted HR of opioid misuse of 1.06 (95% CI, 0.99-1.14) compared with those in the household of a patient who did not receive an opioid prescription. In adjusted models, each additional opioid prescription refill for the patient was associated with a 19.2% (95% CI, 14.5%-24.0%) increase in hazard of opioid misuse in the family member. Each additional week of opioid exposure to the patient was associated with a 12.3% (95% CI, 8.9%-15.8%) increase in hazard of opioid misuse in the family member. Those in households with any refill had a 32.9% (95% CI, 22.7%-43.8%) covariate-adjusted increase in opioid misuse. When the patient became a chronic opioid user (>90 days), the HR for opioid misuse among the family members was estimated to be 2.52 (95% CI, 1.68-3.80). Figure 1 shows all adjusted and unadjusted HR estimates. For brevity and clarity, chronic opioid use results for all exposures are reported in the eResults in the Supplement and broadly follow the patterns of opioid misuse. Results from all Cox proportional hazards regression models are provided in eTables 17 and 18 in the Supplement.

**Figure 2.**
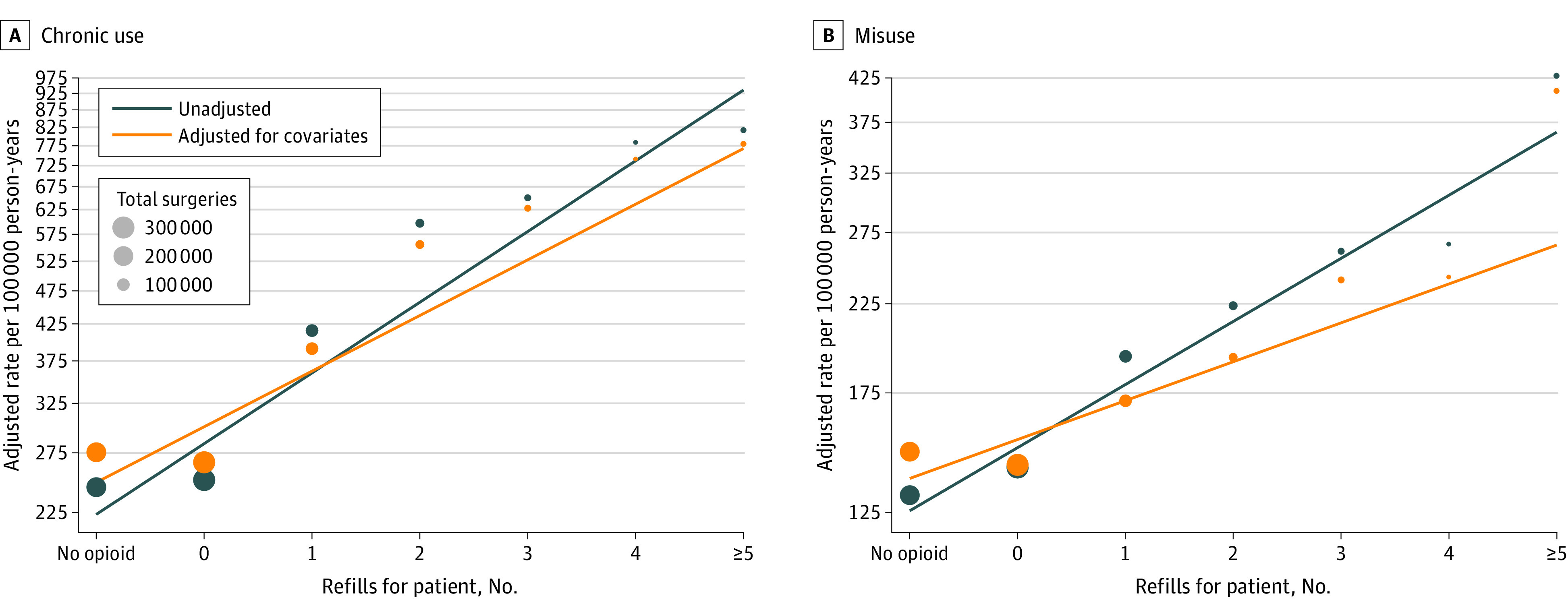
Rate of Opioid Misuse and Chronic Opioid Use in Family Members by Number of Refills for the Patient, Adjusted and Unadjusted for Covariates

**Figure 3.**
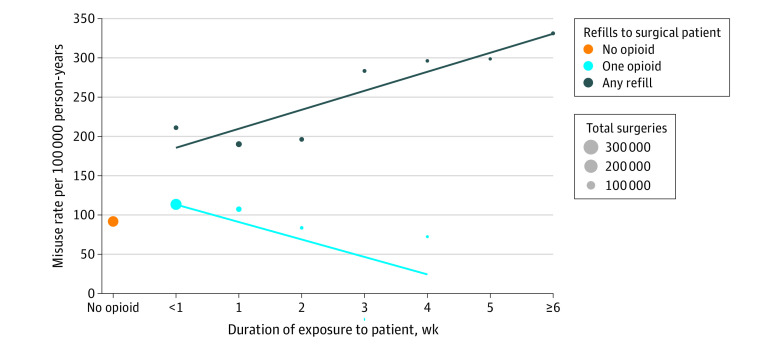
Rate of Opioid Misuse in Family Members by Duration of Exposure and Number of Refills for the Patient

Rates of opioid misuse and chronic opioid use appeared to increase only after a refill was acquired. In Figure 2, the adjusted rate of opioid misuse in a family member is depicted for each refill given to the patient. The rate of opioid misuse among those in households with 1 opioid prescription or 0 refills was nearly identical to the rate among those in households with 0 opioid prescriptions. Even if the patient filled a longer prescription, the initial prescription did not appear to be associated with increased risk among their family members (Figure 3). In households where the patient filled only an initial prescription of longer than 7 days, the adjusted HR for opioid misuse was 0.92 (95% CI, 0.79-1.07) compared with households with 0 opioid prescriptions, and similar results were found for longer prescriptions of 14 days (HR, 0.80; 95% CI, 0.58-1.11).

**Figure 4.**
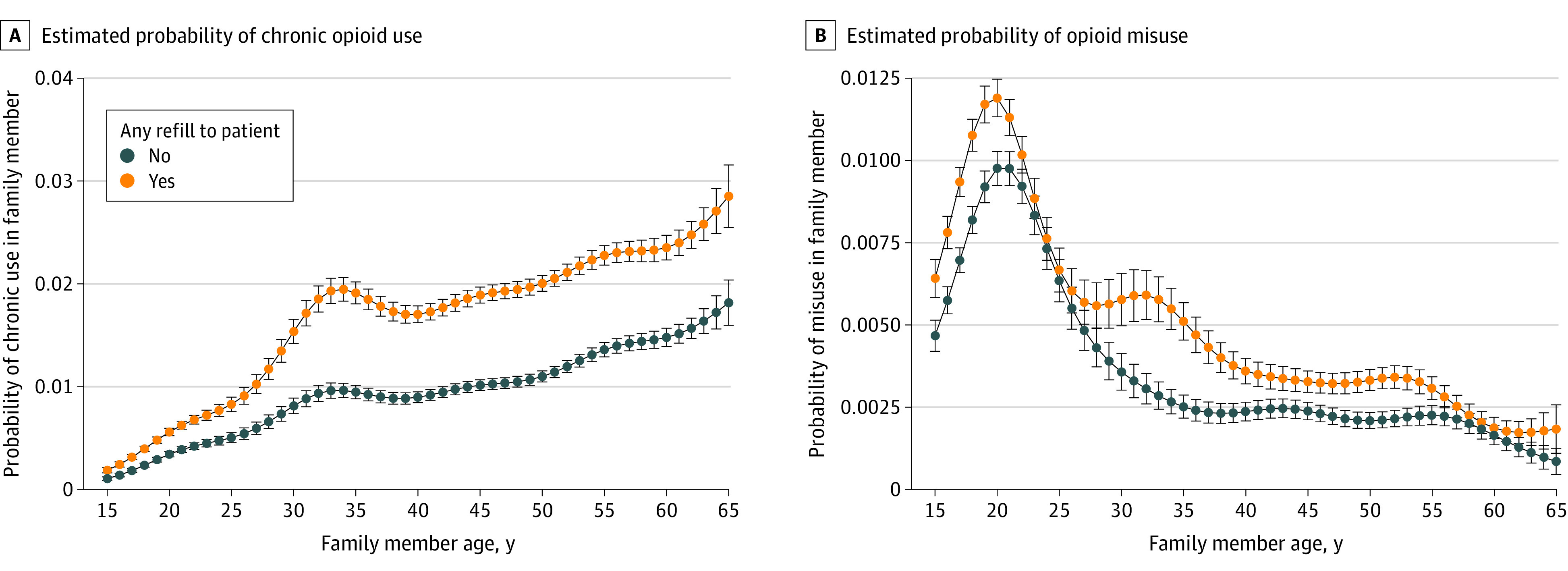
Age-Specific Estimates of Risk of Opioid Misuse and Chronic Opioid Use Among Family Members by Exposure to Refills for the Patient Whiskers indicate 95% CIs.

The probability of opioid misuse and chronic opioid use appeared to diverge in age-specific estimates of risk that compared households with any refill and households with 0 refills (Figure 4). We estimated the highest risk and increase in risk of opioid misuse among younger people, whereas we found the opposite association for chronic opioid use. Risk of chronic opioid use was highest among older family members, whereas risk of opioid misuse was greatest among younger family members (Figure 4).

## Discussion

In a large national cohort of patients and their family members, we found that continued opioid prescribing to the patient was associated with increased risk of both chronic opioid use and opioid misuse among their family members. After adjusting for covariates, filling only an initial prescription without any refills did not appear to be associated with increased risk of opioid misuse or chronic opioid use in family members. There was strong evidence that each refill and additional week of exposure for the patient was associated with increased risks of chronic opioid use and opioid misuse for family members. This finding was strengthened by using multiple outcomes: opioid misuse identified by *ICD-10-CM* diagnosis codes and chronic opioid use identified in pharmacy data. The consistency of effect estimates (Figure 1), despite the outcomes’ different definitions, methods of collection, and age-specific patterns of risk (Figure 4), suggests there may be a durable risk associated with household opioid use. Family members who develop opioid misuse or chronic opioid use are also at an increased risk of illicit opioid use given the established association between prescription opioid abuse and heroin use.^17,18,19,22^

The observed increased risks of opioid misuse and chronic opioid use among family members who were exposed to more opioids in the household can be explained in 3 ways: diversion of unused pills, extended familial exposure, or shared genetic or environmental factors of refills. First, family members may obtain opioids that go unused by the patient. Although we were unable to identify a precise mechanism for these findings, it is possible that diversion of unused pills is a major factor in familial risk. A previous study reported that, in the US, more than 50% of individuals' most recently misused opioids were obtained from friends or family members.^13^ However, we found little evidence that the risk among family members in households with 1 opioid prescription was any higher than the risk in households with 0 opioid prescriptions, despite the likelihood of patients who fill only an initial prescription to have as many or more unused pills than patients who fill a refill, because those with 1 prescription are more likely to take 0 or few pills.^6^ Although this finding does not rule out the possibility of subclinical diversion of pills, findings from this study suggest that initial prescriptions alone do not increase household risk of opioid misuse or chronic opioid use.

Second, familial risk may be triggered by extended exposure to a patient who consumes opioids. Postsurgical opioid exposure has been associated with risk of opioid misuse in the patient.^12^ Exposure, and particularly extended exposure, is more appropriately described as a family exposure whereby susceptible individuals have access to opioids outside of a legitimate prescription. The patient plays a role in the increased risk in their family members.^38^ This pathway could include the sharing of pills among family members rather than diversion of unused pills. A patient with nonmedical use of opioids could request refills because of their continued need or the demand from their family members. Furthermore, addiction in the patient could be associated with the increased risk of addiction in family members.

Third, there may be a shared genetic or environmental factor in both additional refills to the patient and opioid misuse in family members. Genetic makeup,^39^ developmental processes,^40^ and shared environments^41^ are known to play a role in an individual’s susceptibility to addiction. Patients with risky environments or a family predisposition to substance use disorder may be more likely to require or request multiple refills of opioids after surgery. Perceptions and attitudes in the household about opioids and pain control may also be a powerful mechanism because they may make family members more likely to initiate or obtain opioids on their own.^42^ Many opioid risk assessment tools identify a family history of substance use disorder as a risk factor for opioid misuse by a patient.^41^ A combination of genetics and shared environments is a factor in familial aggregation^40^ that is associated with patient refills with family opioid misuse and chronic opioid use.

Further research into these household associations is necessary to clarify their contributions and many other possible mechanisms underlying the findings of this study. Patient-level consumption and reported outcomes data would provide a more granular view of the association between opioid use and misuse as well as some insight into the amount of prescribing prompted by patient opioid requests rather than surgeon prescribing behavior. This study suggests that knowledge of household opioid exposure could be 1 marker to identify patients at higher risk of opioid use and misuse. Interventions to reduce opioid prescribing should consider the possible multiplicative outcomes for both the patient with a prescription and their family members.

This study was not intended to recommend an initial prescription length for prescribers. The results suggest that there are secondary factors in opioid overprescribing. In addition, this study highlights that surgeons should be aware that the risk of opioid misuse may be ambient within a household and that the reasons for multiple prescriptions may include factors beyond ongoing pain. At the same time, given that we do not know the direction of association in this work, clinicians may also be able to explain that extended opioid exposure may have a field effect for patients and their families that should be monitored closely.

### Limitations

This study has several limitations. Administrative claims data provide an incomplete description of patient status, which could lead to the inclusion of patients with undocumented presurgical prescription opioid misuse or chronic opioid use. Furthermore, postsurgical opioid misuse of some family members may go undocumented or may be wrongly documented by the health care system. The clinical fidelity of diagnosis codes in capturing true opioid abuse events has been shown to be suboptimal^43^; for example, overdoses were correctly documented at a rate of only 25%.^44^ Similarly, opioid users may have chronic opioid use patterns that do not meet study definitions or may obtain opioids from other sources. For these reasons, in conjunction with the stringent opioid misuse definition that required an opioid-specific diagnosis, the reported rates of opioid misuse and chronic opioid use should be considered to be conservative lower bounds. Given that rates of opioid misuse are higher among individuals without insurance,^15^ our analysis may also likely be conservative at the national level, which is in line with existing literature that found a similar rate of opioid misuse in a population with commercial insurance.^45,46^ The analysis included only family members aged 15 to 64 years; as such, younger family members or those receiving Medicare may be affected in ways that were not captured. Censoring may have also affected the findings given that patients and family members may have been lost to death, disenrollment, or changing health plans. There may have also been family members in the household who were not captured in the analysis because they did not share an insurance policy.

As a result, we did not attempt to estimate the precise incidence of prescription opioid misuse or chronic opioid use in this population, but we estimated only the association between prescription opioid exposure in patients and prescription opioid misuse and chronic opioid use in their family members. Thus, the importance of these findings is not diminished by the small number of patients included in this study.

Although we attempted to control for confounding by creating balance on observed presurgical diagnoses and prescriptions, there may be relevant patient health factors that went unmeasured. Furthermore, we recognize that patients and families who acquired and then lost insurance coverage within a short period, and thus were excluded from the analysis, may exhibit characteristics that are different from those of the families we were able to track. Accordingly, the generalizability of this study is limited to US families with commercial insurance and consistent coverage.

Several states, pharmacy benefit managers, and commercial payers impose highly variable opioid prescribing restrictions.^47,48^ Although most of these restrictions were implemented at the end of the study period (2017 or later), some were implemented during the study period.^47^ These rules primarily restrict the size and duration of initial opioid prescriptions, but they typically do not restrict refills. Although there is little evidence to suggest that these restrictions have altered initial prescription sizes,^48,49^ it is possible that refill patterns may have experienced variable changes across the US as each new restriction was implemented; for example, refills may be more common in states with more restrictive limits on initial prescription sizes. Given the heterogeneity of these restrictions, variable implementation dates, and unpredictability for individual patients, we did not include these restrictions in this study.

## Conclusions

In this cohort study of patients who received opioids after surgery and their family members, we found that opioid exposure was a household risk. Household risk was observed in patients with at least 1 opioid refill after discharge, and adverse effects of opioid prescriptions extended beyond the patients who received a prescription. Opioid refills after surgery were associated with an increased risk of opioid misuse and chronic opioid use among family members. It is important for prescribers, patients, and the public to understand that opioid misuse is a multifaceted problem that extends beyond the patient and has broader implications for the entire family.

## Notes

Supplement. **eFigure.** CONSORT-Style Data Flow Diagram of Study **eMethods.** Supplementary Statistical Methods **eResults.** Chronic Use Results **eTable 1.** Diagnosis Codes Identifying the Outcome of Opioid Misuse **eTable 2.** Diagnosis Codes Identifying Possible Opioid Misuse Used for Exclusion Based on Pre-surgical Diagnoses **eTable 3.** Raw and Adjusted Rates of Opioid Misuse in Family Members by Any Opioid Exposure to Patient **eTable 4.** Raw and Adjusted Rates of Chronic Opioid Use in Family Members by Any Opioid Exposure to Patient **eTable 5.** Raw and Adjusted Rates of Opioid Misuse in Family Members by Any Opioid Refill to Patient **eTable 6.** Raw and Adjusted Rates of Chronic Opioid Use in Family Members by Any Opioid Refill to Patients **eTable 7.** Raw and Adjusted Rates of Opioid Misuse in Family Members by Number of Opioid Refills to Patients **eTable 8.** Raw and Adjusted Rates of Chronic Opioid Use in Family Members by Number of Opioid Refills to Patients **eTable 9.** Raw and Adjusted Rates of Opioid Misuse in Family Members by Duration of Opioid Exposure to Patient **eTable 10.** Raw and Adjusted Rates of Chronic Opioid Use in Family Members by Duration of Opioid Exposure to Patient **eTable 11.** Raw and Adjusted Rates of Opioid Misuse in Family Members by Chronic Opioid Exposure (>90 Days) to Patient **eTable 12.** Raw and Adjusted Rates of Chronic Opioid Use in Family Members by Chronic Opioid Exposure (>90 Days) to Patient **eTable 13.** Raw and Adjusted Rates of Misuse in Family Members by Refills to Patient: The No-Refill Group Is Stratified by Duration of Less Than 7 Days and At Least 7 Days **eTable 14.** Raw and Adjusted Rates of Chronic Use in Family Members by Refills to Patient: The No-Refill Group Is Stratified by Duration of Less Than 7 Days and At Least 7 Days **eTable 15.** Raw and Adjusted Rates of Misuse in Family Members by Refills to Patient: The No-Refill Group Is Stratified by Duration of Less Than 14 Days and At Least 14 Days **eTable 16.** Raw and Adjusted Rates of Chronic Use in Family Members by Refills to Patient: The No-Refill Group Is Stratified by Duration of Less Than 14 Days and At Least 14 Days **eTable 17.** All Estimated Hazard Ratios for Chronic Use From Inverse-Probability-Weighted Cox Models **eTable 18.** All Estimated Hazard Ratios for Opioid Misuse From Inverse-Probability-Weighted Cox Models
eReferences
