# Supplementary material for: Association of Postsurgical Opioid Refills for Patients With Risk of Opioid Misuse and Chronic Opioid Use Among Family Members
Source: JAMA Netw Open. 2022 Jul 15;5(7):e2221316. doi: 10.1001/jamanetworkopen.2022.21316 (PMC9287751; doi:10.1001/jamanetworkopen.2022.21316)
Supplement: Supplement. — eFigure. CONSORT-Style Data Flow Diagram of Study eMethods. Supplementary Statistical Methods eResults. Chronic Use Results eTable 1. Diagnosis Codes Identifying the Outcome of Opioid Misuse eTable 2. Diagnosis Codes Identifying Possible Opioid Misuse Used for Exclusion Based on Pre-surgical Diagnoses eTable 3. Raw and Adjusted Rates of Opioid Misuse in Family Members by Any Opioid Exposure to Patient eTable 4. Raw and Adjusted Rates of Chronic Opioid Use in Family Members by Any Opioid Exposure to Patient eTable 5. Raw and Adjusted Rates of Opioid Misuse in Family Members by Any Opioid Refill to Patient eTable 6. Raw and Adjusted Rates of Chronic Opioid Use in Family Members by Any Opioid Refill to Patients eTable 7. Raw and Adjusted Rates of Opioid Misuse in Family Members by Number of Opioid Refills to Patients eTable 8. Raw and Adjusted Rates of Chronic Opioid Use in Family Members by Number of Opioid Refills to Patients eTable 9. Raw and Adjusted Rates of Opioid Misuse in Family Members by Duration of Opioid Exposure to Patient eTable 10. Raw and Adjusted Rates of Chronic Opioid Use in Family Members by Duration of Opioid Exposure to Patient eTable 11. Raw and Adjusted Rates of Opioid Misuse in Family Members by Chronic Opioid Exposure (>90 Days) to Patient eTable 12. Raw and Adjusted Rates of Chronic Opioid Use in Family Members by Chronic Opioid Exposure (>90 Days) to Patient eTable 13. Raw and Adjusted Rates of Misuse in Family Members by Refills to Patient: The No-Refill Group Is Stratified by Duration of Less Than 7 Days and At Least 7 Days eTable 14. Raw and Adjusted Rates of Chronic Use in Family Members by Refills to Patient: The No-Refill Group Is Stratified by Duration of Less Than 7 Days and At Least 7 Days eTable 15. Raw and Adjusted Rates of Misuse in Family Members by Refills to Patient: The No-Refill Group Is Stratified by Duration of Less Than 14 Days and At Least 14 Days eTable 16. Raw and Adjusted Rates of Chronic Use in Family Members by Refi [file jamanetwopen-e2221316-s001.pdf]

Supplemental Online Content

Agniel D, Brat GA, Marwaha JS, et al. Association of postsurgical opioid refills for patients with risk of opioid misuse and chronic opioid use among family members. *JAMA Netw Open*. 2022;5(7):e2221316. doi:10.1001/jamanetworkopen.2022.21316

eFigure. CONSORT-Style Data Flow Diagram of Study

eMethods. Supplementary Statistical Methods

eResults. Chronic Use Results

eTable 1. Diagnosis Codes Identifying the Outcome of Opioid Misuse

eTable 2. Diagnosis Codes Identifying Possible Opioid Misuse Used for Exclusion Based on Pre-surgical Diagnoses

eTable 3. Raw and Adjusted Rates of Opioid Misuse in Family Members by Any Opioid Exposure to Patient

eTable 4. Raw and Adjusted Rates of Chronic Opioid Use in Family Members by Any Opioid Exposure to Patient

eTable 5. Raw and Adjusted Rates of Opioid Misuse in Family Members by Any Opioid Refill to Patient

eTable 6. Raw and Adjusted Rates of Chronic Opioid Use in Family Members by Any Opioid Refill to Patients

eTable 7. Raw and Adjusted Rates of Opioid Misuse in Family Members by Number of Opioid Refills to Patients

eTable 8. Raw and Adjusted Rates of Chronic Opioid Use in Family Members by Number of Opioid Refills to Patients

© 2022 Agniel D et al. *JAMA Network Open.*

eTable 9. Raw and Adjusted Rates of Opioid Misuse in Family Members by Duration of Opioid Exposure to Patient

eTable 10. Raw and Adjusted Rates of Chronic Opioid Use in Family Members by Duration of Opioid Exposure to Patient

eTable 11. Raw and Adjusted Rates of Opioid Misuse in Family Members by Chronic Opioid Exposure (>90 Days) to Patient

eTable 12. Raw and Adjusted Rates of Chronic Opioid Use in Family Members by Chronic Opioid Exposure (>90 Days) to Patient

eTable 13. Raw and Adjusted Rates of Misuse in Family Members by Refills to Patient: The No-Refill Group Is Stratified by Duration of Less Than 7 Days and At Least 7 Days

eTable 14. Raw and Adjusted Rates of Chronic Use in Family Members by Refills to Patient: The No-Refill Group Is Stratified by Duration of Less Than 7 Days and At Least 7 Days

eTable 15. Raw and Adjusted Rates of Misuse in Family Members by Refills to Patient: The No-Refill Group Is Stratified by Duration of Less Than 14 Days and At Least 14 Days

eTable 16. Raw and Adjusted Rates of Chronic Use in Family Members by Refills to Patient: The No-Refill Group Is Stratified by Duration of Less Than 14 Days and At Least 14 Days

eTable 17. All Estimated Hazard Ratios for Chronic Use From Inverse-Probability-Weighted Cox Models

eTable 18. All Estimated Hazard Ratios for Opioid Misuse From Inverse-Probability-Weighted Cox Models

eReferences

© 2022 Agniel D et al. *JAMA Network Open.*

This supplemental material has been provided by the authors to give readers additional information about their work.

© 2022 Agniel D et al. *JAMA Network Open.*

eFigure. CONSORT-Style Data Flow Diagram of Study

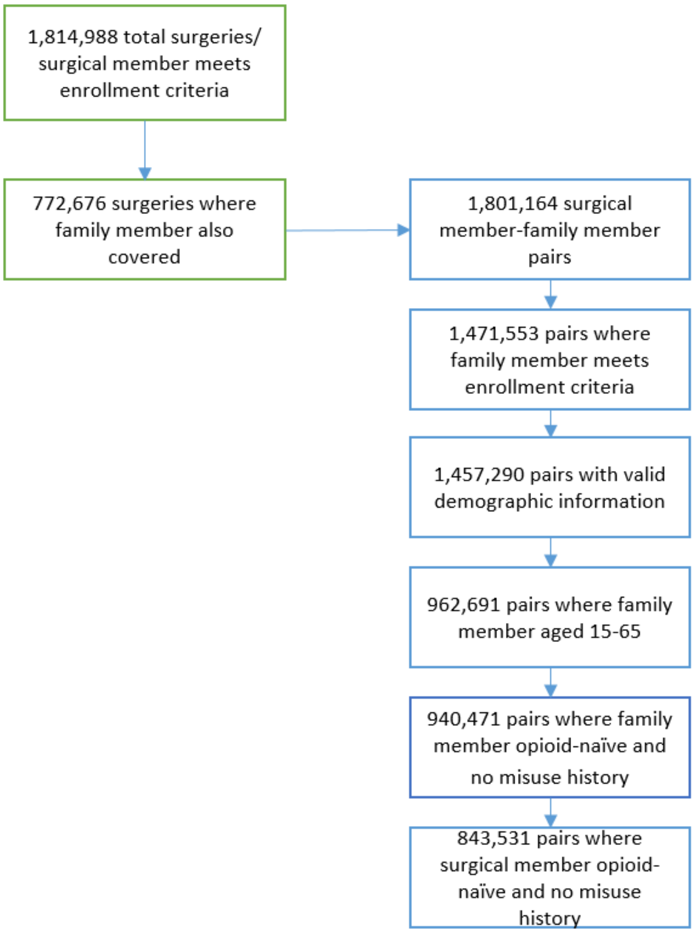

© 2022 Agniel D et al. *JAMA Network Open.*

eMethods. Supplementary Statistical Methods

Inverse probability of treatment weights were used to calculate adjusted estimates of rates of misuse and chronic use (see **Figure 2** and **eTable 3** and following tables) and were used to weight Cox proportional hazards models (similar to Cole and Hernan 2004)[1] to assess the effect of all exposures on misuse and chronic use. Balance of covariates was checked on the weighted dataset, and any covariates that were found to be out of balance (standardized mean difference of more than 0.25 (Stuart, 2010)[2] were included in the Cox models. See **eTable 17** for variables included for all models. Robust sandwich variance estimates were used to account for the fact that members may appear in the dataset multiple times. Weighted generalized additive models (GAMs) were also used to estimate age-specific effects, ignoring follow-up time, via the mgcv package in R. Age-specific covariate balance was checked (see below), and any covariates that were found to be out of balance were included in the GAMs. Follow-up time was ignored for GAM analysis for computational reasons, but analyses including follow-up time were similar to analyses ignoring it (compare **Figure 2** to **eFigure 1** or relative incidences to relative rates in **eTable 3** and following tables). Bonferroni adjustments to p-values made no effect on any interpretations, due to the large sample sizes. More details on the specific steps of the analysis are given below.

Initial covariate selection

We first selected the universe of potential confounders by selecting covariates that were relevant for the outcomes. Models were fit separately for misuse and chronic use. For each outcome, four types of potential confounders were considered: indicators of pre-surgical diagnoses in family members, indicators of pre-surgical diagnoses in patients, measures of prescription drug use in family members, and measures of prescription drug use in patients.

© 2022 Agniel D et al. *JAMA Network Open.*

Penalized logistic regression (lasso) was used to identify important variables of each type. The first model selected predictor diagnoses in family members associated with downstream family member misuse and chronic use. Predictors were selected from all 1,246 diagnoses that occurred in at least 0.05% of family members in the pre-surgical period. A similar lasso regression was run to select from the 2,649 patient diagnoses that occurred in at least 0.05% of patients in the pre-surgical period those that were associated with downstream misuse in family members. Additional models were run to identify important indicators of family member prescription drug use and patient prescription drug use in the pre-surgical period. These covariates corresponded to an indicator of any as well as number and duration of prescriptions filled in 309 prescription drug classes identified via Cerner's Multum Lexicon Drug Database.

Propensity score modeling

Propensity score models were estimated using the lasso to avoid overfitting. In addition to the diagnosis and pharmacy codes identified via the covariate selection above, we also included indicators for each study year (2008-2016) and the relationship to the employee and the sex of both the patient and family member. Traditional propensity scores were used for all binary exposures (any opioid, any refill, and chronic exposure). Duration and refills were treated as multinomial exposures, and multinomial models were used to build generalized propensity scores (Spreeuwenberg et al 2010)[3]. Inverse probability weights were restricted to be less than 100 to ensure that no observations had undue influence on final results.

© 2022 Agniel D et al. *JAMA Network Open.*

Primary analyses

Cox proportional hazards models were estimated for each exposure and outcome. Balance was checked for all exposures by examining standardized mean differences for all potential confounders after weighting. For duration and refills, standardized mean differences were checked for all binarized versions (e.g., 0-2 refills vs 3+ refills, or 0-4 weeks duration vs 5+ weeks duration) of the exposures. Any covariate that was out of balance according to this criterion for any binarized version was included in the final Cox model for that exposure.

Age-specific models

Age-specific models were fit using weighted generalized additive models (GAMs), ignoring follow-up time, via the mgcv package in R. Age-specific covariate balance was checked, and any covariates that were found to be out of balance were included in the GAMs. To check age-specific balance, an additional weight was constructed to compute standardized mean differences among family members of similar ages. This additional weight took the form of a Gaussian kernel, centered at a specific age with standard deviation of 2 years and had the effect of down-weighting any individuals who weren't in a close vicinity of a given age. The following ages were used as kernel centers: 15, 20, 25, …, 60, 65. Any covariate that was out of balance for any age was included in the generalized additive model.

Power Analysis

Due to the large sample size, we have good power to detect even small hazard ratios (HRs). For example, we conservatively estimate in excess of 80% power to detect a HR of 1.11 for the relationship between any opioid and misuse and an HR of 1.08 for the relationship with

© 2022 Agniel D et al. *JAMA Network Open.*

chronic use. Similar power is expected for HRs of 1.13 and 1.09 for the relationship between any refill and misuse and chronic use, respectively. Power was computed using the powerSurvEpi package in R.

© 2022 Agniel D et al. *JAMA Network Open.*

eResults. Chronic Use Results

In this section we report results for the chronic use outcome, which broadly follow the same trends as the misuse outcome reported in the main text. The hazard of chronic use among members in an opioid-exposed household was 1.3 times higher than those not in an opioid-exposed household (CI 1.24-1.36), with an adjusted HR of 1.19 (CI 1.12-1.26). Each refill of opioids filled by the patient was associated with a 22% (CI 19%-25%) increase in hazard of chronic use in the family member on average. Those in households with any refill had a 75% increase in chronic use (CI 65%-85%) after adjusting for covariates. And when patients became chronic users (> 90 days), the HR for chronic use among their family members was estimated to be 3.65 (CI 2.66-5.01). In households where the patient filled a single prescription of longer than 7 days, the adjusted HR for chronic use was 0.99 (CI 0.88-1.12) compared to households where the patient filled no prescription. This HR rose to 1.14 for a single prescription longer than 14 days, but with a wide confidence interval of 0.92-1.42.

© 2022 Agniel D et al. *JAMA Network Open.*

eTable 1. Diagnosis Codes Identifying the Outcome of Opioid Misuse

Diagnosis code	Description
304.0	Opioid type dependence
304.00	Opioid type dependence unspecified use
304.01	Opioid type dependence, continuous use
304.02	Opioid type dependence, episodic use
304.7	Combinations of opioid type drug with any other drug dependence
304.70	Combinations of opioid type drug with any other drug dependence, unspecified use
304.71	Combinations of opioid type drug with any other drug dependence, continuous use
304.72	Combinations of opioid type drug with any other drug dependence, episodic use
305.5	Opioid abuse
305.50	Opioid abuse, unspecified use
305.51	Opioid abuse, continuous use
305.52	Opioid abuse, episodic use
305.53	Opioid abuse, in remission
965	Poisoning by analgesics, antipyretics, and antirheumatics
965.0	Poisoning by opiates and related narcotics
965.00	Poisoning by opium (alkaloids), unspecified
965.09	Poisoning by other opiates and related narcotics
E850.2	Accidental poisoning by other opiates and related narcotics
E935.2	Other opiates and related narcotics causing adverse effects in therapeutic use
F11.10	Opioid abuse, uncomplicated
F11.120	Opioid abuse with intoxication, uncomplicated
F11.121	Opioid abuse with intoxication delirium
F11.122	Opioid abuse with intoxication with perceptual disturbance
F11.129	Opioid abuse with intoxication, unspecified
F11.14	Opioid abuse with opioid-induced mood disorder
F11.150	Opioid abuse with opioid-induced psychotic disorder with delusions
F11.151	Opioid abuse with opioid-induced psychotic disorder with hallucinations
F11.159	Opioid abuse with opioid-induced psychotic disorder, unspecified
F11.181	Opioid abuse with opioid-induced sexual dysfunction
F11.182	Opioid abuse with opioid-induced sleep disorder
F11.188	Opioid abuse with other opioid-induced disorder
F11.19	Opioid abuse with unspecified opioid-induced disorder
F11.20	Opioid dependence, uncomplicated
F11.220	Opioid dependence with intoxication, uncomplicated
F11.221	Opioid dependence with intoxication delirium
F11.222	Opioid dependence with intoxication with perceptual disturbance
F11.229	Opioid dependence with intoxication, unspecified
F11.23	Opioid dependence with withdrawal
F11.24	Opioid dependence with opioid-induced mood disorder
F11.250	Opioid dependence with opioid-induced psychotic disorder with delusions
F11.251	Opioid dependence with opioid-induced psychotic disorder with hallucinations

© 2022 Agniel D et al. *JAMA Network Open.*

F11.259	Opioid dependence with opioid-induced psychotic disorder, unspecified
F11.281	Opioid dependence with opioid-induced sexual dysfunction
**Diagnosis code**	**Description**
F11.282	Opioid dependence with opioid-induced sleep disorder
F11.288	Opioid dependence with other opioid-induced disorder
F11.29	Opioid dependence with unspecified opioid-induced disorder
F11.90	Opioid use, unspecified, uncomplicated
F11.920	Opioid use, unspecified with intoxication, uncomplicated
F11.921	Opioid use, unspecified with intoxication delirium
F11.922	Opioid use, unspecified with intoxication with perceptual disturbance
F11.929	Opioid use, unspecified with intoxication, unspecified
F11.93	Opioid use, unspecified with withdrawal
F11.94	Opioid use, unspecified with opioid-induced mood disorder
F11.950	Opioid use, unspecified with opioid-induced psychotic disorder with delusions
F11.951	Opioid use, unspecified with opioid-induced psychotic disorder with hallucinations
F11.959	Opioid use, unspecified with opioid-induced psychotic disorder, unspecified
F11.981	Opioid use, unspecified with opioid-induced sexual dysfunction
F11.982	Opioid use, unspecified with opioid-induced sleep disorder
F11.988	Opioid use, unspecified with other opioid-induced disorder
F11.99	Opioid use, unspecified with unspecified opioid-induced disorder
T40.0X1A	Poisoning by opium, accidental (unintentional), initial encounter
T40.0X4A	Poisoning by opium, undetermined, initial encounter
T40.0X5A	Adverse effect of opium, initial encounter
T40.2X1A	Poisoning by other opioids, accidental (unintentional), initial encounter
T40.2X4A	Poisoning by other opioids, undetermined, initial encounter
T40.2X5A	Adverse effect of other opioids, initial encounter
T40.4X1A	Poisoning by other synthetic narcotics, accidental (unintentional), initial encounter
T40.4X4A	Poisoning by other synthetic narcotics, undetermined, initial encounter
T40.4X5A	Adverse effect of other synthetic narcotics, initial encounter
T40.601A	Poisoning by unspecified narcotics, accidental (unintentional), initial encounter
T40.604A	Poisoning by unspecified narcotics, undetermined, initial encounter
T40.605A	Adverse effect of unspecified narcotics, initial encounter
T40.691A	Poisoning by other narcotics, accidental (unintentional), initial encounter
T40.694A	Poisoning by other narcotics, undetermined, initial encounter
T40.695A	Adverse effect of other narcotics, initial encounter

© 2022 Agniel D et al. *JAMA Network Open.*

eTable 2. Diagnosis Codes Identifying Possible Opioid Misuse Used for Exclusion Based on Pre-surgical Diagnoses

Diagnosis code	Description
292	Drug-induced mental disorders
292.0	Drug withdrawal
304.0	Opioid type dependence
304.00	Opioid type dependence unspecified use
304.01	Opioid type dependence, continuous use
304.02	Opioid type dependence, episodic use
304.03	Opioid type dependence, in remission
304.7	Combinations of opioid type drug with any other drug dependence
304.70	Combinations of opioid type drug with any other drug dependence, unspecified use
304.71	Combinations of opioid type drug with any other drug dependence, continuous use
304.72	Combinations of opioid type drug with any other drug dependence, episodic use
304.73	Combinations of opioid type drug with any other drug dependence, in remission
304.9	Unspecified drug dependence
304.90	Unspecified drug dependence, unspecified use
304.91	Unspecified drug dependence, continuous use
304.92	Unspecified drug dependence, episodic use
304.93	Unspecified drug dependence, in remission
305.5	Opioid abuse
305.50	Opioid abuse, unspecified use
305.51	Opioid abuse, continuous use
305.52	Opioid abuse, episodic use
305.53	Opioid abuse, in remission
305.53	Opioid abuse, in remission
305.9	Other, mixed, or unspecified drug abuse
305.90	Other, mixed, or unspecified drug abuse, unspecified use
305.91	Other, mixed, or unspecified drug abuse, continuous use
305.92	Other, mixed, or unspecified drug abuse, episodic use
305.93	Other, mixed, or unspecified drug abuse, in remission
965	Poisoning by analgesics, antipyretics, and antirheumatics
965.0	Poisoning by opiates and related narcotics
965.00	Poisoning by opium (alkaloids), unspecified
965.09	Poisoning by other opiates and related narcotics
977.8	Poisoning by other specified drugs and medicinal substances
977.9	Poisoning by unspecified drug or medicinal substance
E850.2	Accidental poisoning by other opiates and related narcotics
E935.2	Other opiates and related narcotics causing adverse effects in therapeutic use
F11.10	Opioid abuse, uncomplicated
F11.120	Opioid abuse with intoxication, uncomplicated
F11.121	Opioid abuse with intoxication delirium

© 2022 Agniel D et al. *JAMA Network Open.*

F11.122	Opioid abuse with intoxication with perceptual disturbance
F11.129	Opioid abuse with intoxication, unspecified
**Diagnosis code**	**Description**
F11.14	Opioid abuse with opioid-induced mood disorder
F11.150	Opioid abuse with opioid-induced psychotic disorder with delusions
F11.151	Opioid abuse with opioid-induced psychotic disorder with hallucinations
F11.159	Opioid abuse with opioid-induced psychotic disorder, unspecified
F11.181	Opioid abuse with opioid-induced sexual dysfunction
F11.182	Opioid abuse with opioid-induced sleep disorder
F11.188	Opioid abuse with other opioid-induced disorder
F11.19	Opioid abuse with unspecified opioid-induced disorder
F11.20	Opioid dependence, uncomplicated
F11.220	Opioid dependence with intoxication, uncomplicated
F11.221	Opioid dependence with intoxication delirium
F11.222	Opioid dependence with intoxication with perceptual disturbance
F11.229	Opioid dependence with intoxication, unspecified
F11.23	Opioid dependence with withdrawal
F11.24	Opioid dependence with opioid-induced mood disorder
F11.250	Opioid dependence with opioid-induced psychotic disorder with delusions
F11.251	Opioid dependence with opioid-induced psychotic disorder with hallucinations
F11.259	Opioid dependence with opioid-induced psychotic disorder, unspecified
F11.281	Opioid dependence with opioid-induced sexual dysfunction
F11.282	Opioid dependence with opioid-induced sleep disorder
F11.288	Opioid dependence with other opioid-induced disorder
F11.29	Opioid dependence with unspecified opioid-induced disorder
F11.90	Opioid use, unspecified, uncomplicated
F11.920	Opioid use, unspecified with intoxication, uncomplicated
F11.921	Opioid use, unspecified with intoxication delirium
F11.922	Opioid use, unspecified with intoxication with perceptual disturbance
F11.929	Opioid use, unspecified with intoxication, unspecified
F11.93	Opioid use, unspecified with withdrawal
F11.94	Opioid use, unspecified with opioid-induced mood disorder
F11.950	Opioid use, unspecified with opioid-induced psychotic disorder with delusions
F11.951	Opioid use, unspecified with opioid-induced psychotic disorder with hallucinations
F11.959	Opioid use, unspecified with opioid-induced psychotic disorder, unspecified
F11.981	Opioid use, unspecified with opioid-induced sexual dysfunction
F11.982	Opioid use, unspecified with opioid-induced sleep disorder
F11.988	Opioid use, unspecified with other opioid-induced disorder
F11.99	Opioid use, unspecified with unspecified opioid-induced disorder
T40.0X1A	Poisoning by opium, accidental (unintentional), initial encounter
T40.0X4A	Poisoning by opium, undetermined, initial encounter
T40.0X5A	Adverse effect of opium, initial encounter
T40.2X1A	Poisoning by other opioids, accidental (unintentional), initial encounter
T40.2X4A	Poisoning by other opioids, undetermined, initial encounter

© 2022 Agniel D et al. *JAMA Network Open.*

T40.2X5A	Adverse effect of other opioids, initial encounter
T40.4X1A	Poisoning by other synthetic narcotics, accidental (unintentional), initial encounter
T40.4X4A	Poisoning by other synthetic narcotics, undetermined, initial encounter
T40.4X5A	Adverse effect of other synthetic narcotics, initial encounter
**Diagnosis code**	**Description**
T40.601A	Poisoning by unspecified narcotics, accidental (unintentional), initial encounter
T40.604A	Poisoning by unspecified narcotics, undetermined, initial encounter
T40.605A	Adverse effect of unspecified narcotics, initial encounter
T40.691A	Poisoning by other narcotics, accidental (unintentional), initial encounter
T40.694A	Poisoning by other narcotics, undetermined, initial encounter
T40.695A	Adverse effect of other narcotics, initial encounter

© 2022 Agniel D et al. *JAMA Network Open.*

eTable 3. Raw and Adjusted Rates of Opioid Misuse in Family Members by Any Opioid Exposure to Patient

Any opioid	n	Misuse events	Incidence, % (95% CI)	Total followup time, years	Misuse rate per 100,000 person-years (95% CI)	Adjusted incidence, % (95% CI)	Adjusted misuse rate per 100,000 person-years (95% CI)
No	303,787	1,210	0.40 (0.38-0.42)	922,175	131 (124-139)	0.44 (0.43-0.45)	147 (143-152)
Yes	539,744	2,684	0.50 (0.48-0.52)	1,649,613	163 (157-169)	0.49 (0.47-0.50)	157 (152-161)

© 2022 Agniel D et al. *JAMA Network Open.*

eTable 4. Raw and Adjusted Rates of Chronic Opioid Use in Family Members by Any Opioid Exposure to Patient

Any opioid	n	Chronic use events	Incidence, % (95% CI)	Total followup time (years)	Chronic use rate per 100,000 person-years (95% CI)	Adjusted incidence (95% CI)	Adjusted chronic use rate per 100,000 person-years (95% CI)
No	303,787	2,253	0.74 (0.71-0.77)	919,750	245 (235-255)	0.79 (0.77-0.81)	266 (260-272)
Yes	539,744	5,232	0.97 (0.94-1.00)	1,644,061	318 (310-327)	0.97 (0.95-0.99)	314 (307-320)

© 2022 Agniel D et al. *JAMA Network Open.*

eTable 5. Raw and Adjusted Rates of Opioid Misuse in Family Members by Any Opioid Refill to Patient

Any refill	n	Misuse events	Incidence, % (95% CI)	Total followup time (years)	Misuse rate per 100,000 person-years (95% CI)	Adjusted incidence, % (95% CI)	Adjusted misuse rate per 100,000 person-years (95% CI)
No	701,205	2,937	0.42 (0.40-0.43)	2,139,854	137 (132-142)	0.44 (0.42-0.45)	144 (139-148)
Yes	142,326	957	0.67 (0.63-0.71)	431,934	222 (208-236)	0.58 (0.57-0.60)	191 (186-196)

© 2022 Agniel D et al. *JAMA Network Open.*

eTable 6. Raw and Adjusted Rates of Chronic Opioid Use in Family Members by Any Opioid Refill to Patients

Any refill	n	Chronic use events	Incidence, % (95% CI)	Total followup time (years)	Chronic use rate per 100,000 person-years (95% CI)	Adjusted incidence (95% CI)	Adjusted chronic use rate per 100,000 person-years (95% CI)
No	701,205	5,301	0.76 (0.74-0.78)	2,134,542	248 (242-255)	0.80 (0.78-0.82)	263 (256-269)
Yes	142,326	2,184	1.5 (1.5-1.6)	429,268	509 (487-530)	1.4 (1.4-1.4)	459 (451-468)

© 2022 Agniel D et al. *JAMA Network Open.*

eTable 7. Raw and Adjusted Rates of Opioid Misuse in Family Members by Number of Opioid Refills to Patients

Refills	n	Misuse events	Incidence, % (95% CI)	Total followup time (years)	Misuse rate per 100,000 person-years (95% CI)	Adjusted incidence, % (95% CI)	Adjusted misuse rate per 100,000 person-years (95% CI)
No opioid	303,787	1,210	0.40 (0.38-0.42)	922,175	131 (124-139)	0.44 (0.43-0.46)	148 (144-153)
0	397,418	1,727	0.44 (0.41-0.46)	1,217,679	142 (135-149)	0.45 (0.43-0.46)	143 (138-148)
1	87,829	519	0.59 (0.54-0.64)	267,430	194 (177-211)	0.53 (0.51-0.54)	171 (166-176)
2	28,265	192	0.68 (0.58-0.78)	85,808	224 (192-255)	0.59 (0.57-0.61)	194 (188-199)
3	12,372	97	0.78 (0.63-0.94)	37,184	261 (209-313)	0.73 (0.71-0.75)	241, (234-247)
4	5,918	47	0.79 (0.57-1.0)	17,659	266 (190-342)	0.73 (0.71-0.76)	243 (234-251)
5+	7,942	102	1.3 (1.0-1.5)	23,853	428 (345-511)	1.2 (1.2-1.3)	410 (400-420)

© 2022 Agniel D et al. *JAMA Network Open.*

eTable 8. Raw and Adjusted Rates of Chronic Opioid Use in Family Members by Number of Opioid Refills to Patients

Refills	n	Chronic use events	Incidence, % (95% CI)	Total followup time (years)	Chronic use rate per 100,000 person-years (95% CI)	Adjusted incidence, % (95% CI)	Adjusted chronic use rate per 100,000 person-years (95% CI)
No opioid	303,787	2,253	0.74 (0.71-0.77)	919,750	245 (235-255)	0.82 (0.80-0.84)	276 (269-282)
0	397,418	3,048	0.77 (0.74-0.79)	1,214,793	251 (242-260)	0.83 (0.81-0.85)	266 (260-273)
1	87,829	1,106	1.26 (1.19-1.33)	266,175	416 (391-440)	1.2 (1.18-1.23)	391 (383-399)
2	28,265	508	1.80 (1.64-1.95)	85,097	597 (545-649)	1.69 (1.66-1.72)	556 (546-565)
3	12,372	240	1.94 (1.70-2.18)	36,897	651 (568-733)	1.89 (1.85-1.92)	628 (617-639)
4	5,918	137	2.32 (1.93-2.70)	17,472	784 (653-915)	2.23 (2.18-2.28)	742 (726-757)
5+	7,942	193	2.43 (2.09-2.77)	23,626	817 (702-932)	2.34 (2.30-2.39)	780 (764-796)

© 2022 Agniel D et al. *JAMA Network Open.*

eTable 9. Raw and Adjusted Rates of Opioid Misuse in Family Members by Duration of Opioid Exposure to Patient

Duration (weeks)	n	Misuse events	Incidence, % (95% CI)	Total followup time (years)	Misuse rate per 100,000 person-years (95% CI)	Adjusted incidence, % (95% CI)	Adjusted misuse rate per 100,000 person-years (95% CI)
0	303,787	1,210	0.40 (0.38-0.42)	922,175	131 (124-139)	0.44 (0.43-0.46)	149 (144-153)
1	367,774	1,672	0.46 (0.43-0.48)	1,135,156	147 (140-154)	0.47 (0.45-0.48)	149 (145-154)
2	101,710	520	0.51 (0.47-0.56)	306,534	170 (155-184)	0.49 (0.47-0.50)	158 (153-163)
3	31,889	174	0.55 (0.46-0.63)	95,357	183 (155-210)	0.50 (0.49-0.52)	163 (158-168)
4	12,038	93	0.77 (0.62-0.93)	35,444	262 (209-316)	0.69 (0.67-0.70)	229 (222-235)
5	9,530	70	0.74 (0.56-0.91)	28,424	246 (189-304)	0.74 (0.72-0.76)	243 (236-250)
6	4,776	41	0.86 (0.60-1.12)	14,059	292 (202-381)	0.82 (0.79-0.85)	276 (267-285)
>6	12,027	114	0.95 (0.77-1.12)	34,639	329 (269-390)	0.85 (0.83-0.87)	288 (280-297)

© 2022 Agniel D et al. *JAMA Network Open.*

eTable 10. Raw and Adjusted Rates of Chronic Opioid Use in Family Members by Duration of Opioid Exposure to Patient

Duration (weeks)	n	Chronic use events	Incidence, % (95% CI)	Total followup time (years)	Chronic use rate per 100,000 person-years (95% CI)	Adjusted incidence, % (95% CI)	Adjusted chronic use rate per 100,000 person-years (95% CI)
0	303,787	2,253	0.74 (0.71-0.77)	919,750	245 (235-255)	0.81 (0.79-0.83)	275 (268-281)
1	367,774	2,975	0.81 (0.78-0.84)	1,132,254	263 (253-272)	0.87 (0.85-0.89)	279 (272-285)
2	101,710	1,091	1.07 (1.01-1.14)	305,267	357 (336-379)	1.06 (1.03-1.08)	343 (336-350)
3	31,889	433	1.36 (1.23-1.49)	94,861	457 (414-500)	1.33 (1.31-1.36)	438 (429-446)
4	12,038	194	1.61 (1.39-1.84)	35,175	552 (474-629)	1.60 (1.57-1.63)	540 (530-550)
5	9,530	174	1.83 (1.56-2.10)	28,209	617 (525-709)	1.73 (1.69-1.76)	572 (561-583)
6	4,776	63	1.32 (1.00-1.64)	14,020	449 (338-560)	1.31 (1.27-1.35)	438 (426-451)
>6	12,027	302	2.51 (2.23-2.79)	34,274	881 (782-981)	2.33 (2.29-2.38)	794 (779-809)

© 2022 Agniel D et al. *JAMA Network Open.*

eTable 11. Raw and Adjusted Rates of Opioid Misuse in Family Members by Chronic Opioid Exposure (>90 Days) to Patient

Chronic exposure	n	Misuse events	Incidence, % (95% CI)	Total followup time (years)	Misuse rate per 100,000 person-years (95% CI)	Adjusted incidence, % (95% CI)	Adjusted misuse rate per 100,000 person-years (95% CI)
No	841,292	3,861	0.50 (0.44-0.50)	2,565,488	151 (146-155)	0.46 (0.45-0.47)	151 (146-156)
Yes	2,239	33	1.50 (0.97-2.00)	6,301	524 (345-702)	1.38 (1.33-1.44)	485 (466-504)

© 2022 Agniel D et al. *JAMA Network Open.*

eTable 12. Raw and Adjusted Rates of Chronic Opioid Use in Family Members by Chronic Opioid Exposure (>90 Days) to Patient

Chronic exposure	n	Chronic use events	Incidence, % (95% CI)	Total followup time (years)	Chronic use rate per 100,000 person-years (95% CI)	Adjusted incidence, % (95% CI)	Adjusted chronic use rate per 100,000 person-years (95% CI)
No	841,292	7,408	0.9 (0.9-0.9)	2,557,584	290 (283-296)	0.9 (0.9-0.9)	290 (284-297)
Yes	2,239	77	3.4 (2.7-4.2)	6,226	1237 (961-1513)	3.3 (3.2-3.4)	1181 (1149-1212)

© 2022 Agniel D et al. *JAMA Network Open.*

eTable 13. Raw and Adjusted Rates of Misuse in Family Members by Refills to Patient: The No-Refill Group Is Stratified by Duration of Less Than 7 Days and At Least 7 Days

Refills	n	Misuse events	Incidence, % (95% CI)	Total followup time (years)	Misuse rate per 100,000 person-years (95% CI)	Adjusted incidence, % (95% CI)	Adjusted misuse rate per 100,000 person-years (95% CI)
No opioid	303,787	1,210	0.40 (0.38-0.42)	922,175	131 (124-139)	0.44 (0.43-0.46)	148 (143-153)
One Rx < 7 days	343,072	1,508	0.44 (0.42-0.46)	1,056,517	145 (136-150)	0.45 (0.44-0.47)	145 (141-150)
One Rx >= 7 days	54,346	219	0.40 (0.35-0.46)	161,162	136 (118-154)	0.42 (0.41-0.44)	136 (131-140)
Any refill	142,326	957	0.67 (0.63-0.71)	431,934	222 (208-236)	0.58 (0.57-0.60)	191 (186-196)

© 2022 Agniel D et al. *JAMA Network Open.*

eTable 14. Raw and Adjusted Rates of Chronic Use in Family Members by Refills to Patient: The No-Refill Group Is Stratified by Duration of Less Than 7 Days and At Least 7 Days

Refills	n	Chronic use events	Incidence, % (95% CI)	Total followup time (years)	Chronic use rate per 100,000 person-years (95% CI)	Adjusted incidence, % (95% CI)	Adjusted chronic use rate per 100,000 person-years (95% CI)
No opioid	303,787	2,253	0.74 (0.71-0.77)	919,750	245 (235-255)	0.81 (0.79-0.83)	273 (266-279)
One Rx < 7 days	343,072	2,617	0.76 (0.73-0.79)	1,054,035	248 (239-258)	0.83 (0.81-0.85)	267 (260-273)
One Rx >= 7 days	54,346	431	0.79 (0.72-0.87)	160,757	268 (243-293)	0.83 (0.81-0.85)	268 (262-275)
Any refill	142,326	2,184	1.54 (1.47-1.60)	429,268	509 (487-530)	1.41 (1.38-1.43)	460 (452-469)

© 2022 Agniel D et al. *JAMA Network Open.*

eTable 15. Raw and Adjusted Rates of Misuse in Family Members by Refills to Patient: The No-Refill Group Is Stratified by Duration of Less Than 14 Days and At Least 14 Days

Refills	n	Misuse events	Incidence, % (95% CI)	Total follow-up time (years)	Misuse rate per 100,000 person-years (95% CI)	Adjusted incidence, % (95% CI)	Adjusted misuse rate per 100,000 person-years (95% CI)
No opioid	303,787	1,210	0.40 (0.38-0.42)	922,175	131 (124-139)	0.44 (0.43-0.46)	148 (143-153)
One Rx < 14 days	386,373	1,687	0.44 (0.42-0.46)	1,184,764	142 (136-149)	0.45 (0.44-0.47)	145 (140-149)
One Rx >= 14 days	11,045	40	0.36 (0.25-0.47)	32,915	122 (84-159)	0.36 (0.35-0.38)	119 (114-123)
Any refill	142,326	957	0.67 (0.63-0.72)	431,934	222 (208-236)	0.58 (0.57-0.60)	190 (185-196)

© 2022 Agniel D et al. *JAMA Network Open.*

eTable 16. Raw and Adjusted Rates of Chronic Use in Family Members by Refills to Patient:

The No-Refill Group Is Stratified by Duration of Less Than 14 Days and At Least 14 Days

Refills	n	Chronic use events	Incidence, % (95% CI)	Total followup time (years)	Chronic use rate per 100,000 person-years (95% CI)	Adjusted incidence, % (95% CI)	Adjusted chronic use rate per 100,000 person-years (95% CI)
No opioid	303,787	2,253	0.74 (0.71-0.77)	919,750	245 (235-255)	0.81 (0.79-0.83)	274 (267-280)
One Rx < 14 days	386,373	2,943	0.76 (0.73-0.79)	1,181,980	249 (240-258)	0.82 (0.80-0.84)	264 (258-271)
One Rx >= 14 days	11,045	105	0.95 (0.77-1.13)	32,813	320 (259-381)	0.95 (0.92-0.97)	310 (303-317)
Any refill	142,326	2,184	1.54 (1.47-1.60)	429,268	509 (487-530)	1.41 (1.38-1.43)	459 (451-468)

© 2022 Agniel D et al. *JAMA Network Open.*

eTable 17. All Estimated Hazard Ratios for Chronic Use From Inverse-Probability-Weighted Cox Models

Included Covariates were found to be out of balance across exposure groups.

Exposure model	Variable	HR	p-value	CI low	CI high
Any opioid	*Any opioid*	*1.190*	*<0.001*	*1.122*	*1.262*
Any opioid refill	*Any opioid refill*	*1.750*	*<0.001*	*1.651*	*1.854*
Each additional opioid fill	*Each additional opioid fill*	*1.222*	*<0.001*	*1.192*	*1.253*
Each additional opioid fill	Age, patient	1.008	0.023	1.001	1.015
Each additional opioid fill	Patient is child of employee	1.445	0.003	1.129	1.849
Each additional week of opioids	*Each additional week of opioids*	*1.155*	*<0.001*	*1.130*	*1.180*
Each additional week of opioids	Age, surgical patient	1.012	<0.001	1.005	1.018
Each additional week of opioids	Patient is child of employee	1.558	<0.001	1.204	2.015
>90 days of opioids	*>90 days of opioids*	*3.649*	*<0.001*	*2.660*	*5.007*
>90 days of opioids	Age, surgical patient	1.015	0.033	1.001	1.028
>90 days of opioids	Patient diagnosis code: 250.00	1.158	0.476	0.774	1.733
>90 days of opioids	Patient diagnosis code: 300.00	1.227	0.471	0.703	2.142
>90 days of opioids	Patient diagnosis code: 305.1	1.370	0.124	0.917	2.047
>90 days of opioids	Patient diagnosis code: 311	1.192	0.525	0.693	2.053
>90 days of opioids	Patient diagnosis code: 401.9	1.177	0.323	0.852	1.628
>90 days of opioids	Patient diagnosis code: 714.0	0.819	0.59	0.397	1.692
>90 days of opioids	Patient diagnosis code: 715.36	0.686	0.454	0.256	1.838
>90 days of opioids	Patient diagnosis code: 715.90	0.896	0.734	0.477	1.686
>90 days of opioids	Patient diagnosis code: 715.96	1.092	0.815	0.525	2.271
>90 days of opioids	Patient diagnosis code: 722.4	1.038	0.93	0.452	2.385
>90 days of opioids	Patient diagnosis code: 722.52	1.139	0.657	0.642	2.019
>90 days of opioids	Patient diagnosis code: 724.2	0.753	0.149	0.511	1.108
>90 days of opioids	Patient diagnosis code: 805.2	1.526	0.655	0.239	9.757
>90 days of opioids	Patient diagnosis code: 805.6	0.019	<0.001	0.002	0.143
>90 days of opioids	Patient diagnosis code: V20.2	0.911	0.697	0.570	1.456
>90 days of opioids	Patient diagnosis code: V43.65	1.927	0.104	0.873	4.256
>90 days of opioids	Patient diagnosis code: V58.69	0.900	0.585	0.617	1.313
>90 days of opioids	Patient diagnosis code: V72.81	1.161	0.38	0.832	1.621
>90 days of opioids	Patient pharmacy claim: Benzodiazepines	1.123	0.602	0.726	1.736
>90 days of opioids	Patient pharmacy claim: Gamma aminobutyric acid analogs	1.285	0.535	0.582	2.836
>90 days of opioids	Patient pharmacy claim: Miscellaneous anxiolytics, sedatives, and hypnotics	0.925	0.756	0.566	1.513
>90 days of opioids	Patient pharmacy claim: Narcotic analgesic combinations	1.206	0.174	0.921	1.580
>90 days of opioids	Patient pharmacy claim: Proton pump inhibitors	1.139	0.552	0.743	1.746
>90 days of opioids	Patient pharmacy claim: Skeletal muscle relaxants	1.101	0.767	0.583	2.077
>90 days of opioids	Patient pharmacy claim: SSRI antidepressants	0.708	0.136	0.450	1.114
>90 days of opioids	Patient is child of employee	1.877	0.011	1.155	3.051
>90 days of opioids	Patient is spouse of employee	0.897	0.415	0.691	1.165

eTable 18. All Estimated Hazard Ratios for Opioid Misuse From Inverse-Probability-Weighted Cox Models

Included covariates were found to be out of balance across exposure groups.

Exposure model	Variable	HR	p-value	CI low	CI high
Any opioid	*Any opioid*	*1.063*	*0.103*	*0.988*	*1.143*
Any opioid refill	*Any opioid refill*	*1.329*	*<0.001*	*1.227*	*1.438*
Each additional opioid fill	*Each additional opioid fill*	*1.192*	*<0.001*	*1.145*	*1.240*
Each additional opioid fill	Age, patient	0.995	0.203	0.986	1.003
Each additional opioid fill	Patient is child of employee	0.941	0.712	0.681	1.301
Each additional week of opioids	*Each additional week of opioids*	*1.123*	*<0.001*	*1.089*	*1.158*
Each additional week of opioids	Age, patient	1.001	0.882	0.992	1.009
Each additional week of opioids	Patient is child of employee	1.204	0.297	0.849	1.707
>90 days of opioids	*>90 days of opioids*	*2.525*	*<0.001*	*1.679*	*3.796*
>90 days of opioids	Age, patient	0.990	0.349	0.970	1.011
>90 days of opioids	Patient diagnosis code: 250.00	1.389	0.293	0.753	2.565
>90 days of opioids	Patient diagnosis code: 300.00	1.178	0.603	0.636	2.182
>90 days of opioids	Patient diagnosis code: 305.1	1.446	0.162	0.862	2.426
>90 days of opioids	Patient diagnosis code: 311	1.424	0.235	0.795	2.548
>90 days of opioids	Patient diagnosis code: 338.29	5.821	<0.001	2.435	13.917
>90 days of opioids	Surgical patient diagnosis code: 518.89	0.904	0.821	0.378	2.162
>90 days of opioids	Patient diagnosis code: 715.90	1.629	0.352	0.583	4.549
>90 days of opioids	Patient diagnosis code: 786.05	1.162	0.598	0.665	2.028
>90 days of opioids	Patient diagnosis code: 959.8	3.652	0.011	1.350	9.880
>90 days of opioids	Patient diagnosis code: V20.2	0.569	<0.001	0.429	0.754
>90 days of opioids	Patient pharmacy claim: Benzodiazepines	1.866	0.023	1.090	3.195
>90 days of opioids	Patient pharmacy claim: Gamma aminobutyric acid analogs	0.427	0.04	0.190	0.960
>90 days of opioids	Patient pharmacy claim: Miscellaneous antiemetics	0.858	0.767	0.310	2.372
>90 days of opioids	Patient pharmacy claim: Miscellaneous anxiolytics, sedatives, and hypnotics	1.423	0.262	0.768	2.635
>90 days of opioids	Patient pharmacy claim: Narcotic analgesic combinations	1.620	0.321	0.625	4.196
>90 days of opioids	Patient pharmacy claim: Opioids	0.728	0.499	0.291	1.824
>90 days of opioids	Patient pharmacy claim: Skeletal muscle relaxants	0.703	0.462	0.274	1.800
>90 days of opioids	Patient is child of employee	0.625	0.174	0.318	1.230
>90 days of opioids	Patient is spouse of employee	0.517	<0.001	0.370	0.721

© 2022 Agniel D et al. *JAMA Network Open.*

eReferences

1. Cole SR, Hernán MA. Adjusted survival curves with inverse probability weights. *Comput Methods Programs Biomed*. 2004. doi:10.1016/j.cmpb.2003.10.004
2. Stuart EA. Matching methods for causal inference: A review and a look forward. *Stat Sci*. 2010. doi:10.1214/09-STS313
3. Spreeuwenberg MD, Bartak A, Croon MA, et al. The multiple propensity score as control for bias in the comparison of more than two treatment arms: An introduction from a case study in mental health. *Med Care*. 2010. doi:10.1097/MLR.0b013e3181c1328f

© 2022 Agniel D et al. *JAMA Network Open.*
